# Role of common human TRIM5α variants in HIV-1 disease progression

**DOI:** 10.1186/1742-4690-3-54

**Published:** 2006-08-22

**Authors:** Valérie Goldschmidt, Gabriela Bleiber, Margaret May, Raquel Martinez, Millàn Ortiz, Amalio Telenti, The Swiss HIV Cohort Study

**Affiliations:** 1 Institute of Microbiology and University Hospital, University of Lausanne, Switzerland; 2 Department of Social Medicine, University of Bristol, UK

## Abstract

**Background:**

The retroviral restriction factor tripartite motif protein (TRIM)5α, is characterized by marked amino acid diversity among primates, including specific clusters of residues under positive selection. The identification of multiple non-synonymous changes in humans suggests that TRIM5α variants might be relevant to retroviral pathogenesis. Previous studies have shown that such variants are unlikely to modify susceptibility to HIV-1 infection, or the course of early infection. However, the longterm effect of carrying Trim5α variants on disease progression in individuals infected with HIV-1 has not previously been investigated.

**Methods:**

In a cohort of 979 untreated individuals infected with HIV-1 with median follow up 3.2 years and 9,828 CD4 T cell measurements, we analysed common amino acid variations: H43Y, V112F, R136Q, G249D, and H419Y. The rate of CD4 T cell decline before treatment was used as the phenotype. In addition, we extended previous work on the *in vitro *susceptibility of purified donor CD4 T cells (n = 125) to HIV-1 infection, and on the susceptibility of HeLa cells that were stably transduced with the different *TRIM5 *variants. Haplotypes were analysed according to the most parsimonious evolutionary structure, where two main human TRIM5α groups can be defined according to the residue at amino acid 136. Humans present both Q136 and R136 at similar frequency, and additional TRIM5α amino acid variants are almost exclusively derived from R136-carrying haplotypes.

**Results:**

We observed modest differences in disease progression for evolutionary branches carrying R136-derived haplotypes, and with the non-synonymous polymorphisms G249D and H419Y. *In vitro *analysis of susceptibility of donor CD4 T cells, and of the various transduced HeLa cell lines supported the absence of significant differential restriction of HIV-1 infection by the various huTRIM5α alleles.

**Conclusion:**

Common human variants of TRIM5α have no effect or modest effect on HIV-1 disease progression. These variants occur at sites conserved throughout evolution, and are remote from clusters of positive selection in the primate lineage. The evolutionary value of the substitutions remains unclear.

## Background

The tripartite motif (TRIM) family is a well conserved family of proteins characterized by a structure comprising a RING domain, one or two B-boxes and a predicted coiled-coil region [1]. In addition, most TRIM proteins have additional C-terminal domains. Members of the TRIM protein family are involved in various cellular processes, including cell proliferation, differentiation, development, oncogenesis and apoptosis (for recent review [2,3]). Some TRIM proteins display antiviral properties, targeting retroviruses in particular [4].

TRIM5α is a retroviral restriction factor targeting the early steps of cellular infection [4]. TRIM5α restricts retroviral infection by specifically recognizing the capsid and promotes its premature disassembly [5]. Human TRIM5α (huTRIM5α) has limited efficacy against HIV-1, while some primate TRIM5α orthologues can potently restrict this particular lentivirus (for review see [2,3]). Considerable inter-species sequence diversity characterizes TRIM5α and might underlie differences in the pattern and breadth of restriction of multiple lentiviruses. Evolutionary analysis reveals that up to 2% of codons of TRIM5α are predicted to be under positive selection with high confidence [6,7]. Residues under positive selection cluster in the C-terminal B30.2 domain. A first cluster resides between amino acids 322 to 340 in the variable region v1 [7,8], a region previously described as a "patch" of positive selection [6]. Replacement of the v1 region, or of specific amino acids within v1, modifies the restriction pattern of TRIM5α [9,10]. The second cluster, localized between amino acids 381 to 389 [7], corresponds to the previously described variable region v2 of the B30.2 domain [8]. Substitution of the human v2 region by a v2 from Rhesus monkeys exhibits no inhibitory activity against HIV-1 [9,10]. However, v2 variants are thought to result in species-specific lentiviral restriction patterns [11]. An additional region of considerable variation among Sooty mangabeys and Rhesus monkeys has been mapped to the coiled-coil motif [12].

Recently, two studies have addressed the potential role of huTRIM5α variants in modulating susceptibility to HIV-1 [13,14]. Sawyer et al. identified several non-synonymous SNPs in huTRIM5α, but only one of these (H43Y) was found to have a functional consequence [13]. H43Y lies in the RING domain of TRIM5α and may negatively affect its putative E3 ubiquitin ligase activity. Although huTRIM5α weakly restricts HIV-1, H43Y might further reduce viral restriction to a level similar to that of cells expressing no exogenous huTRIM5α [13]. To assess whether the impaired retroviral restriction seen with exogenously expressed huTRIM5α H43Y resulted in altered susceptibility in human cells, Sawyer et al. tested B-lymphocytes from four individuals: one homozygous for H43, two homozygous for 43Y, and one heterozygous at this residue. Challenge with HIV-1 failed to demonstrate a significant effect of the H43Y change, although 43Y homozygous cells could be infected with N-MLV about 100-fold more efficiently than cells with the common H43 allele [13]. In a second study, Speelmon et al. assessed the association of various non-synonymous variants with susceptibility to HIV-1 among 110 HIV-1 infected subjects and 96 exposed seronegative persons [14]. This study identified possible associations between specific haplotypes and alleles and susceptibility to infection and viral setpoint after acute infection.

In our study, we analysed data from a large cohort of subjects infected with HIV-1 to explore whether different huTRIM5α variants are associated with long-term disease evolution. The study is complemented by analysis of CD4 cell susceptibility to HIV-1, and the *in vitro *functionality of selected huTRIM5α variants. We conclude from our study that there is no impact for some and negligible to modest impact for other common human variants of TRIM5α on disease progression.

## Results and discussion

### TRIM5α polymorphism

**Figure 1:**
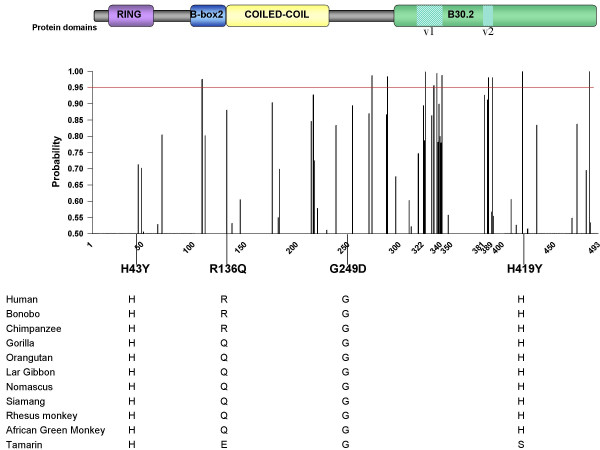
**Position of common human TRIM5α amino acid variants in the context of primate sequence conservation and of the v1 and v2 patches of positive pressure**. Y-axis: posterior probabilities of positively selected codons. X-axis: human TRIM5α amino acid numbering. The evolutionary analysis is adapted from reference [7].

TRIM5α is characterized by important sequence diversity in humans, as shown in this and in previous studies [13,14]. Analysis of huTRIM5α polymorphism in blood donors identified 21 genetic variants (Additional file 1), including four SNPs leading to non-synonymous changes: 127C>T (rs3740996, H43Y, allelic frequency *f *= 0.11), 407G>A (rs10838525, R136Q, *f *= 0.35), 12468G>A (rs11038628, G249D, *f *= 0.08), and 15142C>T (rs28381981, H419Y, *f *= 0.05). One additional common variant (rs11601507, V112F, *f *= 0.08), and several rare non-synonymous variants have been described in the other studies [13,14]. Changes involve evolutionary conserved positions(Figure 1). None of these changes are within patches of positive selection in primates, in particular none are in the proximity of variable regions v1 and v2 of the B30.2 domain (Figure 1), and thus their evolutionary significance is uncertain. We speculate that R136Q might result from balancing selection because humans carry Q136, the ancestral amino acid, and R136, an amino acid shared only with chimpanzees, with similar frequencies.

### Association of genetic variants with *in vitro *susceptibility to infection

**Figure 2:**
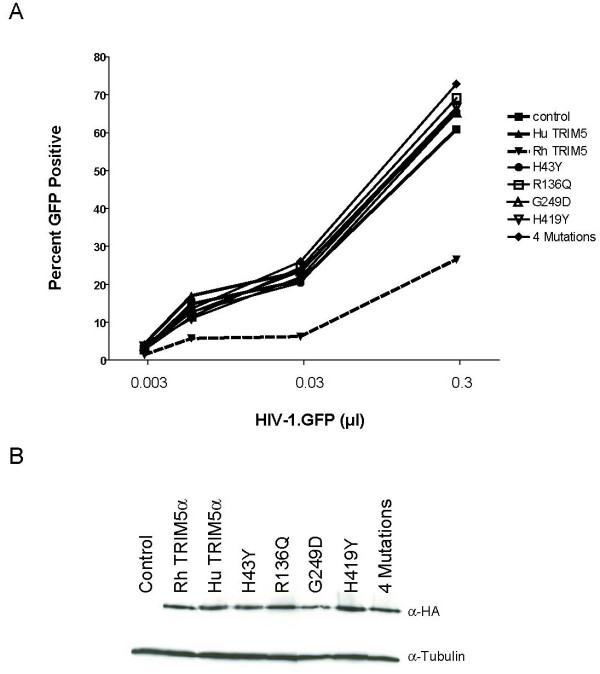
**Restriction of HIV-1 by common human TRIM5α variants**. HeLa cells were stably transduced by oncoretroviral vectors expressing the Rhesus (Rh) TRIM5α, the common huTRIM5α and its variants, separately or in a hypothetical four-mutation protein. Panel A: Single-cycle infectivity assays used VSV-pseudotyped recombinant viruses (HIV.1-GFP) at various m.o.i. After 48 h, cells were analysed by fluorescence-activated cell sorter, and scored for number of GFP-positive cells. Panel B: Expression of HA-tagged TRIM5α proteins was determined by western blotting using an anti-HA antibody. Tubulin was detected with the anti-α tubulin antibody. Control: HeLa cells transduced with an empty oncoretroviral vector.

Data from Sawyer et al. indicated that H43Y results in reduced capacity to restrict N-MLV, but has minimal or no impact on HIV-1 susceptibility to infection *in vitro *in feline fibroblasts (CRFK) cells [13]. We extend and confirm these results by showing that HeLa cells stably transduced with the different human variants of *TRIM5 *do not differ in susceptibility to HIV-1 infection (Figure 2). We also confirmed that the H43Y variant failed to restrict N-MLV in the HeLa background (Additional file 2). Thus, results in Hela cells, that express TRIM5α endogenously, are in full concordance with data from CRFK cells. HeLa cells have the most common TRIM5 alleles (-2CC, H43, V112, heterozygous R136Q, G249, H419).

Speelmon et al. [14] reported on the permissiveness of purified CD4 T cells from 77 seronegative donors. Analysis included assessment of H43Y, R136Q, H419Y, and a series of less common variants. We performed similar experiments by infecting purified CD4 T cells from 125 Caucasian healthy blood donors with replicating HIV-1. Alleles tested included H43Y, R136Q, H419Y, and the common variant G249D (not tested in the above study). There was no significant association of specific variants or haplotypes with *in vitro *p24 production after 7 days (Additional file 3). None of the additional SNPs investigated *in vitro *were associated with differences in cell permissiveness (Additional file 1).

### Association of genetic variants with disease progression *in vivo*

The reports by Sawyer et al and Speelmon et al. [13,14] suggested that some of the alleles could indeed have an impact on HIV-1 susceptibility *in vivo*. We extended their analyses by investigating the association of the various huTRIM5α variants with long-term disease progression in a large cohort of individuals infected with HIV-1. The clinical phenotype was defined as the patient-specific rate of CD4 T cell decline, a recognized marker of disease progression [15]. Analysis excluded any CD4 T cell values after initiation of treatment. The median follow up time was 3.2 years, during which 979 cohort participants, not receiving antiretroviral treatment, contributed 9,828 CD4 T cell determinations to the analysis (median 7 CD4 T cell determinations per participant). We first tested for associations of individual non-synonymous polymorphisms with differences in the natural history of disease progression. H43Y and R136Q had no effect on disease progression. Participants who had one or two copies of G249D or H419Y had slower progression although confidence intervals were wide due to small numbers. Compared to non-carriers who had a square root transformed CD4 gradient of -2.02, participants who were carriers of G249D and H419Y had gradients of -1.74 (95% CI -1.39 to -2.09, p = 0.11) and -1.64 (95% CI -1.33 to -1.95, p = 0.02) respectively.

**Figure 3:**
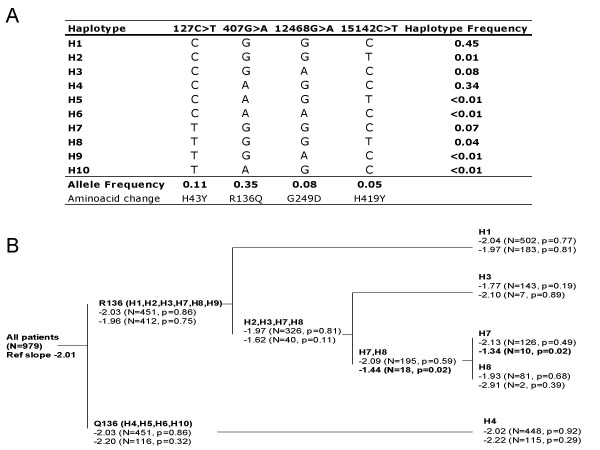
**Association of human TRIM5α haplotypes with HIV-1 disease progression *in vivo***. Panel A, Inferred haplotypes carrying non-synonymous variants. Panel B, Analysis of haplotypes according to the most parsimonious evolutionary analysis, where two main huTRIM5α groups can be defined according to the residue at codon 136. Shown are square root CD4 gradient (reference slope for all patients is -2.01). Top set of slope estimates corresponds to one copy of haplotype(s) group, bottom set is for two copies of haplotype(s) group. Only the H7 haplotype presented a slope significantly different from that of all patients (uncorrected p value). However, p values did not reach the experiment-wide significance of 0.003 (Simes modified Bonferroni p value).

Haplotypes were assessed according to the most parsimonious evolutionary analysis, where two main huTRIM5α groups can be defined according to the residue at amino acid 136 (Figure 3). With the notable exception of chimpanzees and humans that carry an arginine at position 136, all other primates code for a glutamine at codon 136 (glutamic in tamarins), which therefore represents the ancestral sequence for old world monkeys, gibbons and apes. However, humans have similar frequencies of Q136 and R136, and additional TRIM5α amino acid variants are almost exclusively derived from R136-carrying haplotypes (data from this study and from [14]). We did not observe differences in HIV-1 disease progression for evolutionary branches carrying Q136- or R136-containing haplotypes (Figure 3). Weak associations of some haplotypes with disease progression were found, but the p values did not reach the experiment wide corrected significance level of p = 0.0028 (Simes modified Bonferroni p value).

Whilst our study was being completed, Sawyer et al. [13] and Speelmon et al [14] reported on an additional common variant V112F (allelic frequency of 7%). We re-genotyped the cohort and identified the presence of 112F in R136-carrying haplotypes. We confirm the absence of significant effect of this particular amino acid on disease progression [CD4 T cell gradient: -2.21 (95% CI -1.89 to -2.534) compared to mean reference slope -2.01 ]. In addition, Speelmon et al. included in their analysis the 5'UTR -2C>G SNP [14]. The -2C represents the ancestral allele and is present in high linkage disequilibrium with Q136. Speelmon et al. identified a rare haplotype, where individuals carried -2G in the context of Q136, possibly associated with susceptibility to HIV infection (OR 5.49, p = 0.02) [14]. We also identified this rare haplotype (frequency of 1.4%), but did not observe any association with disease progression [CD4 T cell gradient: -2.04 (95% CI -1.20 to -2.88)].

## Conclusion

We have extended previously reported findings on huTRIM5α by showing that the amino acid variant H43Y which results in loss of restriction of N-MLV [13] is not associated with significant differences in HIV-1 disease progression in a large human cohort. The present study also underscores the modest or negligible effect of human variants G249D and H419Y and of some haplotypes on HIV-1 susceptibility and disease progression. Despite the conserved nature of these residues in primates, the evolutionary relevance of the variants in humans is uncertain. However, it is possible that the polymorphisms found in TRIM5α might have been selected in past epidemics by viruses unrelated to HIV-1.

## Materials and methods

### Cells

CD4 T cells from 125 healthy Caucasian blood donors were isolated by anti-CD4 magnetic beads (Miltenyi Biotech) and cultured *ex vivo *in RPMI-1640 (Gibco-Invitrogen), supplemented with 20% fetal calf serum (FCS), 20 U/ml human IL-2 (Roche) and 50 μg/ml gentamicin, following stimulation with 2 μg/ml phytohaemagglutinin (PHA) during two days. CD4 T cells (10^6 ^cells) were infected with R5 clone HIV-1 NL4-3BaL*env *(1000 pg p24 antigen) for 2 hours at 37°C, 5% CO_2_, in 1 ml final volume. Cells were washed and cultured for 7 days. Virus-containing supernatant was harvested and p24 antigen production was monitored by ELISA (Abbott). Permissiveness was defined as the ability of cells to be infected and sustain replication of HIV-1 [16]. The *ex vivo *viral replication for each genotype was represented by the median p24 antigen production at day 7.

### Identification of SNPs, and allelic discrimination

Single nucleotide polymorphism (SNP) discovery used single strand conformation polymorphism and sequencing of 94 chromosomes (47 Caucasian blood donors). For this, a total of 21 PCR reactions were designed to cover exons, putative promoter regions, and intron-exon boundaries (6771 bp/subject). SNPs resulting in non-synonymous substitutions were then genotyped by using TaqMan allelic discrimination (Additional file 4).

### Biological analysis of huTRIM5α variants

The pLPCX oncoretroviral vector containing the human and Rhesus *TRIM5α *gene with an HA epitope tag was obtained from the NIH AIDS Reagent Program (donated by J. Sodroski). Variants of huTRIM5α were made by using the QuikChange protocol (Stratagene). Retroviral vectors were packaged by co-transfecting the various pLPCX constructs with the pNB-tropic MLV Gag-Pol and pVSV-G packaging plasmids [17]. Supernatants were concentrated and used to transduce HeLa cells. Seventy two hours after transduction, cells were selected in 0.5 mg/ml puromycin. Expression of HA-tagged TRIM5α proteins were determined by Western blotting using an anti-HA antibody (Roche). Tubulin was detected with the anti-α tubulin antibody (Sigma). Single-cycle infectivity assays in HeLa cells used the VSV-pseudotyped recombinant viruses HIV.1-GFP and N-MLV-GFP at various m.o.i. Cells were analysed by fluorescence-activated cell sorter (FACS) 48 h after transduction.

### *In vivo *analysis: CD4 cell count decline

Study participants (n = 979) were recruited within the genetics project of the Swiss HIV Cohort Study [18]. The ethics committees of all participant centers approved the study. Patients gave written informed consent for genetic testing. DNA from PBMCs was used for genotyping. Their characteristics are shown in Additional file 5. The rate of decline in CD4 T cell count during the natural history of disease progression was defined as study phenotype as previously reported [19]. The CD4 T cell trajectories were modeled using a repeated measures hierarchical approach using Mlwin software [20]. Square root transformed CD4 T cell counts were modeled as a linear function of time since estimated date of seroconversion with random effects for both the intercept and the gradient with additional terms for sex, age, and risk group [19]. For each genotype, the average square root CD4 decline per year was estimated in dominant and recessive models. Haplotypes were attributed using PHASE [21].

## Competing interests

The author(s) declare that they have no competing interests.

## Authors' contributions

VG and GB carried out re-sequencing, genotyping studies, and construction of expression vectors. VG performed the transduction assays. MM did statistical analyses, data modeling and revised the manuscript. RM did SNP discovery and genotyping studies. MO performed the bioinformatics analyses. AT conceived the study, supervised the molecular genetic analysis, secured funding, and drafted the manuscript.

## Supplementary Material

Additional file 1 Genetic variants and their association with HIV-1 cell permissiveness *in vitro *in purified CD4 T cells from 125 healthy blood donors. Click here for file

Additional file 2 Restriction of N-MLV by common human TRIM5α variants. Click here for file

Additional file 3 Analysis of association of specific human TRIM5α variants or haplotypes and *in vitro *p24 production 7 days post infection of purified CD4 T cells from healthy blood donors with an R5-tropic viral strain. Click here for file

Additional file 4 TaqMan allelic discrimination primers and probes. Click here for file

Additional file 5 Characteristics of 979 subjects infected with HIV-1. Click here for file
