# Supplementary material for: Role of common human TRIM5α variants in HIV-1 disease progression
Source: Retrovirology. 2006 Aug 22;3:54. doi: 10.1186/1742-4690-3-54 (PMC1560158; doi:10.1186/1742-4690-3-54)
Supplement: Additional file 1 — Genetic variants and their association with HIV-1 cell permissiveness in vitro in purified CD4 T cells from 125 healthy blood donors. [file 1742-4690-3-54-S1.pdf]

Additional file 1. Genetic variants and their association with HIV-1 cell permissiveness *in vitro* in purified CD4 T cells from 125 healthy blood donors. 11= common allele homozygous, 12= heterozygous, 22= rare allele homozygous.

TRIM5 region	Base change (Amino Acid)	Rs number	p24 (pg/ml)			P value
			11	12	22	
5' Regulatory region	-5116A/G	rs3802981	78775	78495	89830	0.8671
	-4904T/C[a]	rs3802980	57042	90140	84700	0.8952
Untranslated exon 1	-4811T/C[a]	rs28381978	84700	169660[b]		
Exon 2	127C/T (H43Y)	rs3740996	80500	89830	157590[b]	0.4486
	407G/A (R136Q)	rs10838525	89830	76025	80350	0.8893
Intron 2	615A/G	NR	NA	NA	NA	NA
	759C/T	rs10769175	77250	84160	142150[b]	0.1245
	777C/T	rs28381979	81740	88955	38711[b]	0.3204
Exon 3	1068G/A	rs3740995	81120	84160	157590[b]	0.8635
Intron 3	1358T/A	rs28381980	84700	84290	38711[b]	0.3891
	1607T/G	rs3740994	81120	78490	159295[b]	0.1718
Exon 5	12468G/A (G249D)	rs11038628	77245	104070	187050[b]	0.1452
Exon 8	15142C/T (H419Y)	rs28381981	80500	95430	52955[b]	0.7106
Untranslated exon 8	15632C/A	NR	NA	NA	NA	NA
	15655C/A	NR	NA	NA	NA	NA
	15789G/T	NR	NA	NA	NA	NA
	15794C/A	NR	NA	NA	NA	NA
	15823A/G	NR	NA	NA	NA	NA
	16001C/A	NR	NA	NA	NA	NA
	16023G/A	NR	NA	NA	NA	NA
	16521T/C	rs6578672	76000	100085	97970[b]	0.4883

[a] 94 chromosomes   [b] ≤ 5 participants   NR : not reported   NA : not analysed
