# Supplementary material for: Role of common human TRIM5α variants in HIV-1 disease progression
Source: Retrovirology. 2006 Aug 22;3:54. doi: 10.1186/1742-4690-3-54 (PMC1560158; doi:10.1186/1742-4690-3-54)

**Additional file 2.** Restriction of N-MLV by common human TRIM5α variants. HeLa cells were stably transduced by oncoretroviral vectors expressing the common huTRIM5α and its variants, separately or in a hypothetical four-mutation protein. Single-cycle infectivity assays used VSV-pseudotyped recombinant viruses (N.MLV.GFP). After 48 h, cells were analysed by fluorescence-activated cell sorter (FACS), and scored for number of GFP-positive cells.

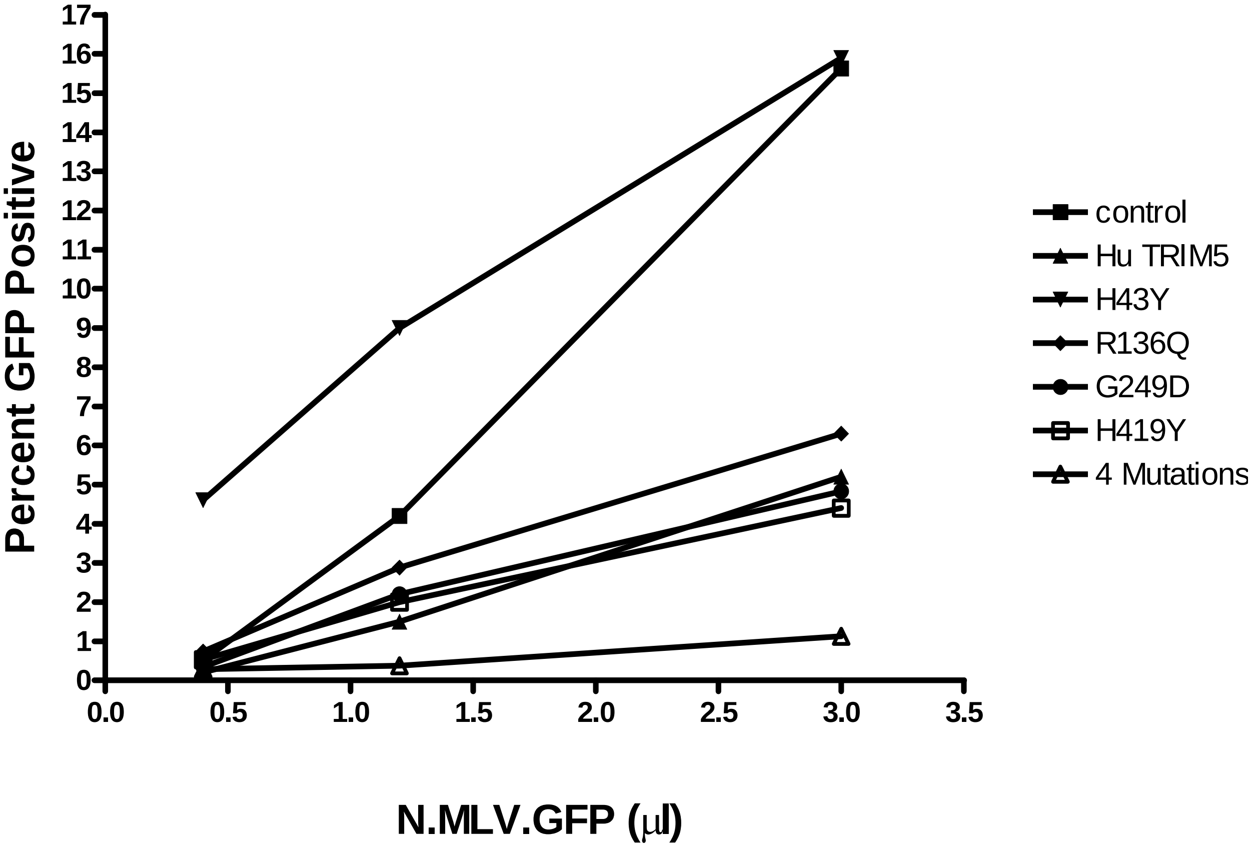

Supplement: Additional file 2 — Restriction of N-MLV by common human TRIM5α variants. [file 1742-4690-3-54-S2.pdf]
