# Supplementary material for: Role of common human TRIM5α variants in HIV-1 disease progression
Source: Retrovirology. 2006 Aug 22;3:54. doi: 10.1186/1742-4690-3-54 (PMC1560158; doi:10.1186/1742-4690-3-54)

Additional file 3. Analysis of association of specific TRIM5α variants (A-D) or haplotypes (E) with *in vitro* p24 production 7 days post infection of purified CD4 T cells from healthy blood donors with R5 tropic HIV-1 NL4-3BaL*env*. The apparent difference between infection in CD4 T cells carrying the common haplotypes H1 and H4, and of CD4 T cells carrying one or more copies of H2 was not statistically significant; median 76.000 vs. 37000 pg/ml, p=0.2.

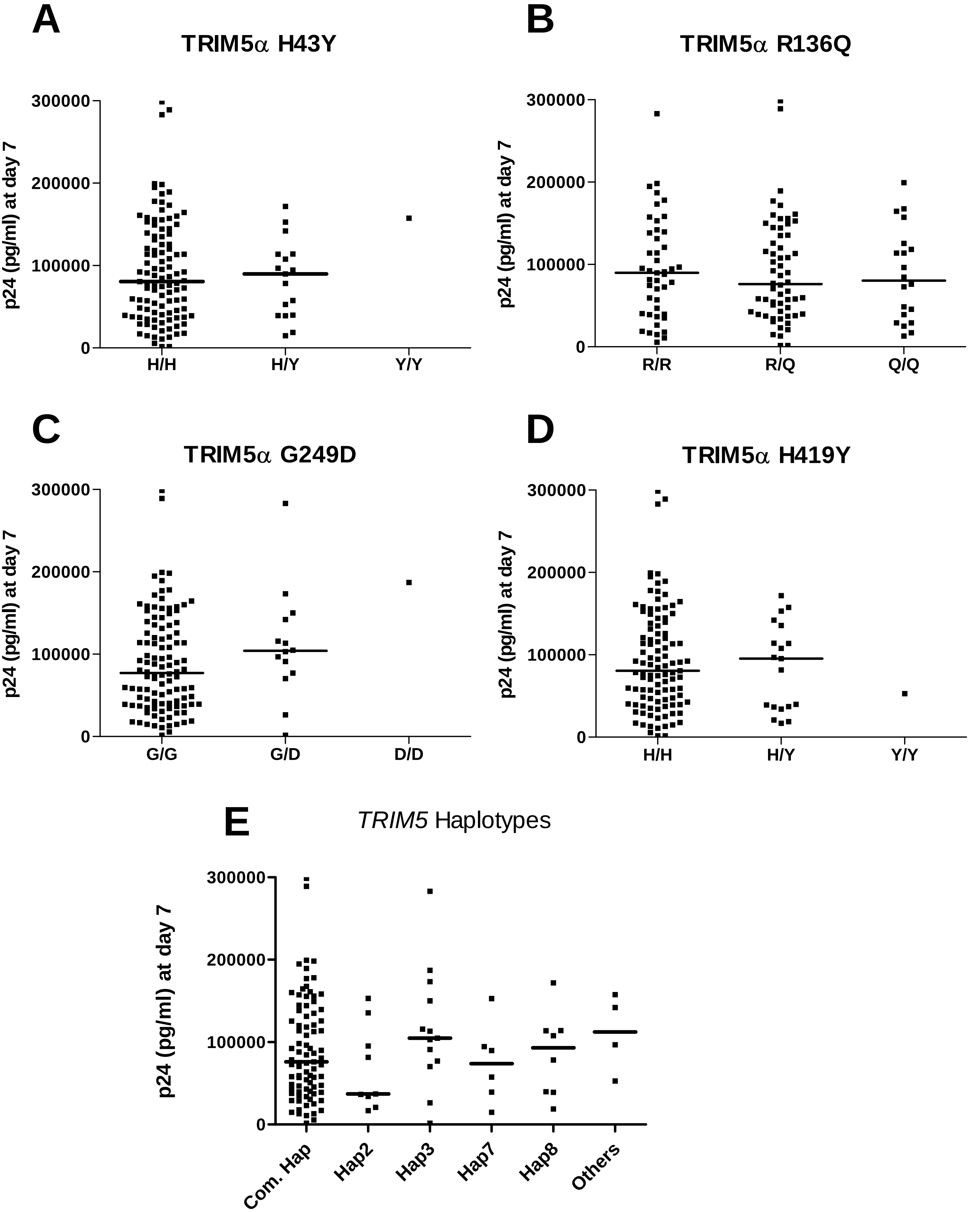

Supplement: Additional file 3 — Analysis of association of specific human TRIM5α variants or haplotypes and in vitro p24 production 7 days post infection of purified CD4 T cells from healthy blood donors with an R5-tropic viral strain. [file 1742-4690-3-54-S3.pdf]
