# Supplementary material for: Role of common human TRIM5α variants in HIV-1 disease progression
Source: Retrovirology. 2006 Aug 22;3:54. doi: 10.1186/1742-4690-3-54 (PMC1560158; doi:10.1186/1742-4690-3-54)
Supplement: Additional file 4 — TaqMan allelic discrimination primers and probes. [file 1742-4690-3-54-S4.pdf]

Additional file 4. TaqMan allelic discrimination primers and probes.

Primers and Probes designation	5'->3' Sequence	Strand
Forward primer -2C>G	CAGGGATCTGTGAACAAGAGGAA	(+)
Reverse primer -2C>G	GTCACCTCCTCCTTTACATTAACCA	(-)
FAM probe -2C>G	ATAGCTAcTATGGCTTC	(+)
VIC probe -2C>G	ATAGCTAgTATGGCTTC	(+)
Forward primer H43Y	CCACAGCTTCTGCCAAGCAT	(+)
Reverse primer H43Y	CTACTCTCTCCTTTGTCTAGCATGGA	(-)
FAM probe H43Y	ACTGCAAACcACAAGA	(+)
VIC probe H43Y	ACTGCAAACtACAAGAA	(+)
Forward primer V112F	GAAGCTCAGGGAGGTCAAGTTG	(+)
Reverse primer V112F	GACCACGGTGCTCCTGAGA	(-)
FAM probe V112F	ACGGGAAGgTCATTT	(+)
VIC probe V112F	ACGGGAAGtTCATTT	(+)
Forward primer R136Q	TCACCACACGTTCCTCACAGA	(+)
Reverse primer R136Q	CTCTCTTCCTTCCATCCCAGTCT	(-)
FAM probe R136Q	TTACTTGGTACTCCcGG	(-)
VIC probe R136Q	TTACTTGGTACTCCtGGG	(-)
Forward primer G249D	AGGAGAGACTCTTTCTTTCTTAATTGATG	(+)
Reverse primer G249D	TGTCCTCCCACACATACCTTTTT	(-)
FAM probe G249D	ATCCACAcCCTAGGAA	(-)
VIC probe G249D	CATCCACAtCCTAGGAA	(-)
Forward primer H419Y	GTTAGAGGAAGGAGTTAAATGTAGTGCTT	(+)
Reverse primer H419Y	GGGCACAATGAAAGGAACAGA	(-)
FAM probe H419Y	TTCCTTCcATACTCC	(+)
VIC probe H419Y	AGTTCCTTCtATACTCC	(+)
