# Supplementary material for: Role of common human TRIM5α variants in HIV-1 disease progression
Source: Retrovirology. 2006 Aug 22;3:54. doi: 10.1186/1742-4690-3-54 (PMC1560158; doi:10.1186/1742-4690-3-54)
Supplement: Additional file 5 — Characteristics of 979 subjects infected with HIV-1. [file 1742-4690-3-54-S5.pdf]

Additional file 5. Characteristics of 979 HIV-1 infected individuals.

Characteristic	No. (%)
Female	286/979 (29%)
IDU	279/979 (29%)
	**Median (inter-quartile range)**
Age at registration (years)	32 (27 to 39)
Follow up time (years)	3.2 (0.8 to 6.1)
No. of CD4 measurements per patient	7 (3 to 13)
First measured CD4 count (cells/μL)	390 (200 to 650)

IDU = intravenous drug use
